# Optimization of Municipal Waste Collection Routing: Impact of Industry 4.0 Technologies on Environmental Awareness and Sustainability

**DOI:** 10.3390/ijerph16040634

**Published:** 2019-02-21

**Authors:** Tamás Bányai, Péter Tamás, Béla Illés, Živilė Stankevičiūtė, Ágota Bányai

**Affiliations:** 1 Institute of Logistics, University of Miskolc, 3515 Miskolc, Hungary; alttpeti@uni-miskolc.hu (P.T.); altilles@uni-miskolc.hu (B.I.); altagota@uni-miskolc.hu (Á.B.); 2 School of Economics and Business, Kaunas University of Technology, 44249 Kaunas, Lithuania; zivile.stankeviciute@ktu.lt

**Keywords:** collection, cyber-physical system, emission reduction, environmental awareness, heuristic optimization, Industry 4.0, Internet of Things, logistics process, waste collection

## Abstract

The accelerated movement of people towards cities led to the fact that the world’s urban population is now growing by 60-million persons per year. The increased number of cities’ population has a significant impact on the produced volume of household waste, which must be collected and recycled in time. The collection of household waste, especially in downtown areas, has a wide range of challenges; the collection system must be reliable, flexible, cost efficient, and green. Within the frame of this paper, the authors describe the application possibilities of Industry 4.0 technologies in waste collection solutions and the optimization potential in their processes. After a systematic literature review, this paper introduces the waste collection process of downtowns as a cyber-physical system. A mathematical model of this waste collection process is described, which incorporates routing, assignment, and scheduling problems. The objectives of the model are the followings: (1) optimal assignment of waste sources to garbage trucks; (2) scheduling of the waste collection through routing of each garbage truck to minimize the total operation cost, increase reliability while comprehensive environmental indicators that have great impact on public health are to be taken into consideration. Next, a binary bat algorithm is described, whose performance is validated with different benchmark functions. The scenario analysis validates the model and then evaluates its performance to increase the cost-efficiency and warrant environmental awareness of waste collection process.

## 1. Introduction

Today, 54% of the world’s population lives in urban areas. This proportion is expected to increase to 66% until 2050 [1]. This intensive increase of the world’s population leads to increased waste production. The waste management systems can be divided into two main parts: technological part and logistics. We focus on logistics related aspects of waste management system, especially from collection point of view within the frame of this paper.

**Figure 1:**
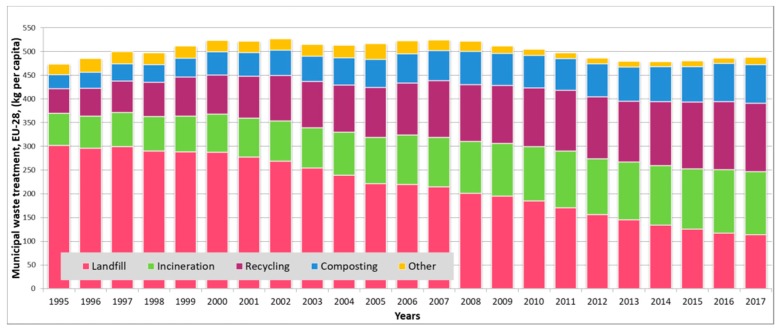
Trends of municipal waste treatment in European Union-28 (EU-28). The data source from [7].

There are a wide range of waste treatment technologies, including anaerobic digestion, biodrying [2], gasification [3], dumping, landfarming, composting, pyrolysis [4], sewage treatment [5], incineration [6], and reuse, but some of them have a huge environmental impact and they can cause serious environmental pollution. The disposal of waste can generate greenhouse gases absorbing and emitting radiant energy within the thermal infrared range and cause the greenhouse effect. Incineration technology can also cause environmental problems, because the incineration of plastic can generate toxic substances, like hydrocarbons, sulfur dioxide, hydrogen chloride, or nitrogen oxides. It means that available technologies must be developed to decrease the generation of pollutants. In previous years, the proportion of used waste treatment technologies has changed. As Figure 1 shows, the proportion of landfill is significantly decreased, while the proportion of incineration, recycling, and composting, as more environmentally friendly waste treatment technologies, have increased.

The incineration technology is a widely spread waste treatment technology, because it can be used, not only for mixed municipal waste, but also for pretreated municipal waste, hazardous waste, sewage sludge, and clinical waste [8]. Within the frame of our model, we are focusing on waste collection processes that are related to incineration-based waste treatment technologies.

**Figure 2:**
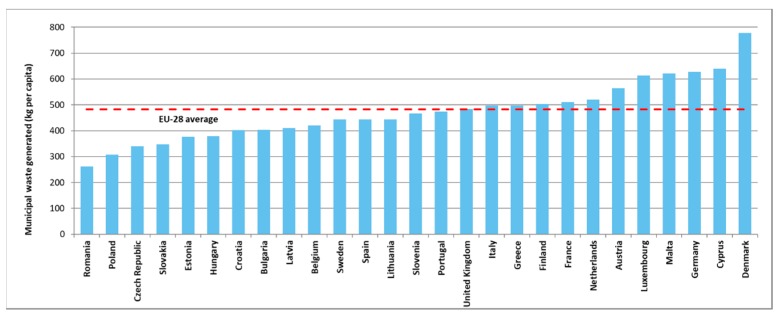
Municipal waste generated by country in 2016. The data source from [7].

The development of waste management systems has been influenced by policymakers. They are responsible for the enforcement and monitoring of regulations, laws, and directives. As Figure 2 shows, the average generated waste in European Union-28 (EU-28) is close to 500 kg per capita. This value means that waste management systems must collect and process a huge amount. The collection and treatment of this huge amount of waste have significant impact on the public health, because collection and treatment processes are causing environmental pollution [9], like the emission of CO_2_, SO_2_, CO, HC, and particulate matter (PM) [10].

The motivation of this paper is that Industry 4.0 technologies make it possible to transfer conventional manufacturing and service systems into cyber-physical systems. Within the frame of our paper, we would like to analyze the impact of Industry 4.0 technologies on the optimization possibilities of waste collection systems, especially from an energy usage and greenhouse gas (GHG) emission point of view. The objective of our paper is to describe a cyber-physical waste collection system, including smart solutions and demonstrating the optimization possibilities in a cyber-physical waste collection system focusing on routing problems.

This paper is organized, as follows. Section 2 presents a literature review, which systematically summarizes the research background of design of waste management systems. Section 3 describes the model framework of cyber-physical waste management system, including Industry 4.0 technologies. Section 4 presents a binary bat algorithm. For our study, in Section 5, we focus on the optimization results with numerical analysis. The discussions and future research directions are discussed in Section 6, while conclusions are in Section 7.

## 2. Literature Review

Within the frame of this chapter, the following questions are answered with a systematic literature review: Who is doing what? Who first did it or published it? What are research gaps?

### 2.1. Conceptual Framework and Review Methodology

Our used methodology of systematic literature review includes the following aspects [11]: formulate of research questions, select sources from Web of Science, reduce the number of articles by reading them and identify the main topic, define a methodology to analyze the chosen articles, describe the main scientific results, and identify the scientific gaps and bottlenecks.

Firstly, the relevant terms were defined. It is a crucial phase of the review, because there are excellent review articles in the field of waste management, and we did not want to produce an almost similar review. We used the following keywords to search in the Web of Science database: TOPIC: (“waste” and “logistics” and “collection”). Initially, 240 articles were identified. This list was reduced to 227 articles, selecting journal articles only. Our search was conducted in November 2018; therefore, new articles may have been published since then.

### 2.2. Descriptive Analysis

**Figure 3:**
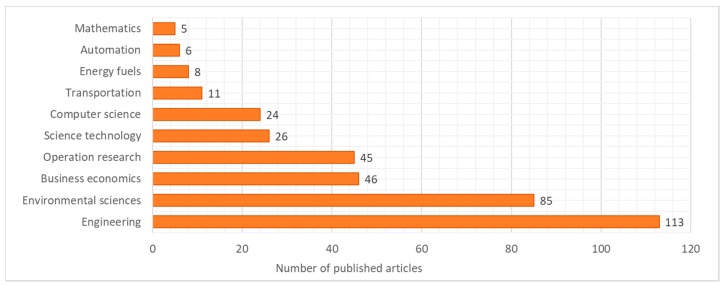
Classification of articles considering subject areas based on search in Web of Science database using TOPIC: “waste” AND “logistics” AND “collection”.

The reduced articles can be classified depending on the research area. Figure 3 shows the classification of these 227 articles while considering 10 subject areas. This classification shows the majority of engineering and environmental sciences, while the operational research and computer sciences define the importance of computational methods that are related to the design of municipal waste collection solutions.

**Figure 4:**
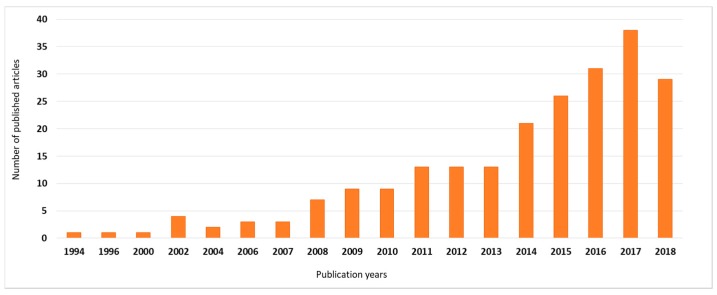
Classification of articles by year of publication based on search in Web of Science.

As Figure 4 demonstrates, the design and operation problems of waste collection systems from a logistics point of view have been researched in the past 20 years. The first article in this field was published in 1994 in the field of inventory management [12] and it was focusing on the “inventory is waste” philosophy in just-in-time production processes. The number of published papers has been increased in the last 10 years; it shows the importance of this research field.

**Figure 5:**
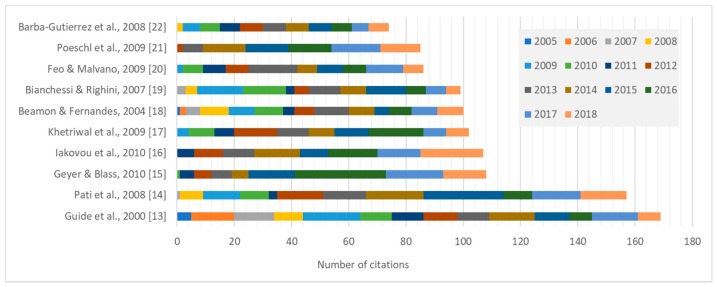
The 10 most cited articles based on search in Web of Science [13,14,15,16,17,18,19,20,21,22].

Articles were analyzed from scientific impact point of view. The most usual form to evaluate articles from a scientific impact point of view is the citation. Figure 5 shows the 10 most cited articles with their number of citations.

**Figure 6:**
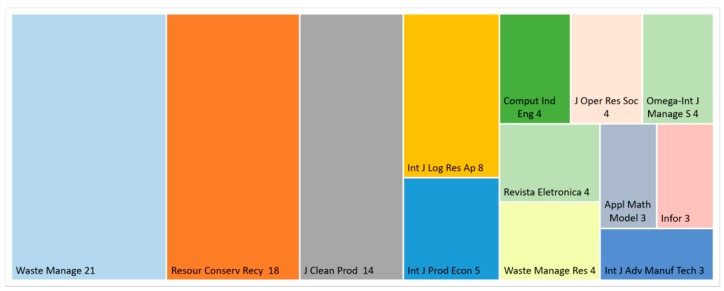
Distribution of waste collection related articles in journals, based on search in Web of Science.

As Figure 6 demonstrates, most of the articles were published in journals with waste management and cleaner technologies topics, but a significant part of the papers was accepted for the publication in journals focusing on computation, operation research, and mathematics. The distribution of journals shows that the design and operation problems of waste management systems are a multidisciplinary problem, where not only technological, but also economic and other aspects, must be taken into consideration.

**Figure 7:**
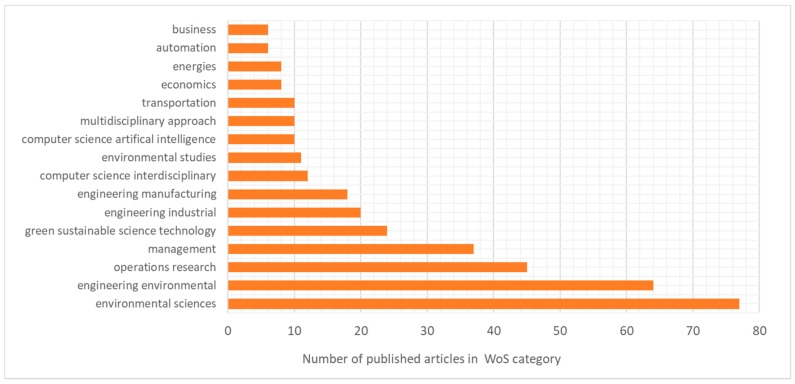
Distribution of papers according to Web of Science categories.

We have analyzed the published articles from a Web of Science categories point of view. We have analyzed the distribution of articles in the following categories: environmental sciences, engineering environment, operations research, management sciences, green sustainable science technologies, industrial engineering, manufacturing engineering, computer science, environmental studies, artificial intelligence in computation, multidisciplinary approach, transportation, economics, energy, automation, and business. The distribution of the categories is depicted in Figure 7. As the categories show, the design of waste collection systems is based on optimization methods, and not only cost efficiency, but also environmental, technological, and social aspects are important.

In the following step, the initial 227 articles were reduced after reading them. We excluded articles where the topic did not arouse our interest and could not be addressed the design of municipal waste collection systems focusing on environmental awareness and public health aspects. After this reduction, we attained 45 articles.

### 2.3. Content Analysis

The literature introduces a wide range of methods that are used to solve design and operation problems of waste management systems, like integer programming, decision-making methods, heuristic and metaheuristic algorithms, Petri Net simulation, statistical approaches, simulation and simulation-based optimization, fuzzy goal programing, or empirical studies. An analytical hierarchy process (AHP)-based multicriteria decision-making model was used to evaluate the costs and business relations for critical decisions regarding the network design of reverse logistics, with a focus on location decision [23]. Integer linear programming, like mixed integer linear programming or multi-period integer programming, makes it possible to analyze and evaluate waste management systems. Researchers used mixed integer goal programming (MIGP) to analyze the inter-relationship between multiple objectives of a recycled paper distribution network to determine the facility location, route, and waste flow in a multi-item, multi-echelon, and multi-facility decision-making framework [14]. The multi-period integer programming model was used to support the joint analysis of investment and operational costs in a closed loop supply chain network, including waste-related processes [18]. In the case of an end-of-life vehicle recovery system in Ankara, the network design, including the different actors, was performed and supported by a mixed integer linear programming (MILP) model [24]. The design problem of two-echelon municipal waste management system for glass, paper, plastic, and organic material can be described as a complex set covering and maximum satisfiability problem, which was solved for the metropolitan area of Barcelona with a genetic algorithm (GA) and greedy randomized adaptive search procedure (GRASP) [25]. The periodic vehicle routing problem with intermediate facilities (PVRP-IF) of municipal waste collection of glass, metal, plastics, and paper was solved with a hybrid solution method that was based on variable neighborhood search (VNS) and dynamic programming [26]. Researchers described and compared constructive algorithms, like local search algorithms and tabu search algorithms with arc-exchange-based and node-exchange-based neighborhoods, while employing different and interacting tabu lists to solve the vehicle routing problem with integrated goods distributions and the waste collection supply chain [19]. The mentioned optimization methods make the optimization of the waste management system from logistics and technology point of view possible. For example, it is possible to calculate the optimal ratio of different available treatment technologies, like incineration, composting, and landfill [27]. In the case of stochastic parameters and uncertain environment, fuzzy logic-based methods, like the interval fuzzy possibilistic model, fuzzy goal programming, or fuzzy colored Petri net simulation can be used. The integrated forward and reverse supply chain networks have a wide range of uncertain parameters, and to find a robust network design in the case of the iron and steel industry, a bi-objective interval fuzzy possibilistic chance-constraint mixed integer linear programming was used [28]. The main objective of the application of a fuzzy goal programming method was to optimize a multi-objective, multi-echelon, multi-product, and multi-period closed loop supply chain as an uncertain network optimization model [29]. Researchers used the fuzzy colored Petri net forecasting method (FCPN) that focused on facility location problems to simulate the uncertain processes of action model of end-of-life products [30]. Hybrid algorithm combining metaheuristics with simulation is a suitable tool to analyze the different collection problems of multiple depots and stochastic parameters [31].

Several scenarios and case studies that were related to different types of waste collection and waste management problems were assessed and evaluated in order to compare the effects of technology, logistics, human resources, policies, and social aspects. Case studies show that the collection of municipal waste, electrical and electronic equipment, and end-of-life vehicles depend on the optimal structure of waste management system, where, among other things, the recovery network, collection, and disposal process must be analyzed and then optimized. A Portugal case study shows that the generic MILP model can provide support to the strategic expansion plans of waste management companies [32], especially from recovery network design point of view. A three-phase hierarchical approach was proposed to optimize the design of the reverse logistics network in the Spanish region of Galicia, with a focus on facility location problem, fleet vehicle routing problem, and collection routing problems [33]. A real case study that was performed in Ankara described the complexity of end-of-life vehicle supply chain problems, especially from the suggested new end-of-life vehicle recovery system point of view [24]. The impact of uncertain information is demonstrated with a Hong Kong case study, where the difficulties of the design of appropriate infrastructure for waste collection and recycling are described. The study shows that the e-waste trading sector increases this uncertainty, and therefore the development of producer responsibility scheme is an import milestone for the increased efficiency of collection [34]. As a demonstrated in a Denmark case study, the design and control of waste management systems must be efficient and fair for all partners, including end users, customers, producers, municipalities, recycling, and collection companies [35]. In developed countries, the realization of the whole waste management system from the implementation of waste management directives to the operation of collection and recovery networks was realized in short time, but increased awareness would lead to a more environmentally sound behavior [36]. In the Industry 4.0 environment, the waste management systems apply increasing IoT technologies to improve the efficiency and reliability of the collection and reuse. Geographic Information System tools are suggested to support the definition of the best waste collection routes, suitable vehicle fleet, and logistic and technological capacity to be used by the Kampala Capital City Authority [37]. The integration of RFID technologies into the collection and recovery processes can increase the efficiency and availability of the whole waste management system [38], while intelligent e-containers are used in Italy to improve the efficiency of logistic processes [39]. A simulation-based case study in Taiwan investigated the factors that affect the design of a collection channel for waste lead-acid storage batteries. The results of the simulation study show that the analysis and optimization of different scenarios of the waste management system increase its cost efficiency, reliability, and performance [40].

The applied mathematical models of waste management systems represent a wide range of approaches, like vehicle routing, facility location, bin allocation, network modelling, or center location. Various applications of vehicle routing problems are described in the literature: the branch-and-price technique is used to optimize the capacitated routing problem of simultaneous distribution and collection [41], tabu search heuristic is used to redesign the collection routes and then compares the collection options of plastic waste using eco-efficiency as a performance indicator [42], and the heuristic algorithm is used to solve a multi-trip vehicle routing problem [43]. Special models of routing problems are the routing problems with split loads and date window [44], the design of periodic routing problems for waste vegetable oil collection [45], or the multi-objective, multi-depot periodic vehicle routing problem with inter-depot routes [46]. Routing is an important issue in waste management, but in practice, the choice of vehicle and waste container type also matters, because the performance of waste collection systems and the related costs and emissions are influenced by the choice of use of trucks, trailers, or comprimator trucks. However, in many cases, the routing takes place with an existing set of trucks, but fleet management and investment in different types of vehicles is also an important component of waste collection optimization. The facility location represents a wide range of waste management problems, because the collection of municipal wastes is performed in a large geographical area, where various supply chain objects, like the collection areas of municipal waste and the treatment sites for recycling, composting, or incinerating, must be located [25]. The facility location problems are usually integrated with the determination of required capacities because the location of collection centers influences the required collection and pre-treatment capacities [47]. However, the facility location is generally focused on the objects of collection system, but the determination of the optimal location for recycling stations and plants is also an important part of the optimal waste management system design, as illustrated with a case study in Beijing, China [48].

Waste management systems are placed in a dynamic environment, where the impact of the environment on the performance of waste collection and treatment can be analyzed while using system dynamics. Researchers used system dynamics to simulate the performance of closed-loop supply chains and then investigated a variety of supply chain solutions [49]. The integrated approach of waste management systems can be represented in many ways, and a multi-objective multi-period multi-product multi-site aggregate production planning model is presented in a green supply chain while considering a reverse logistics network. The integrated approach includes recyclability, biodegradability, energy consumption, and product risk aspects [50]. In the case of integrated design, the multicriteria optimization model can be solved with various models, from multiple objective linear programming to artificial intelligence-based metaheuristic algorithms. A linear programming application led to decreased logistics costs, consumption of fossil fuel, and production of emissions due to transportation [51]. Another approach of integrated design model is the combination of bin allocation, bin sizing, and location, which is solved with a combination of an effective variable neighborhood search metaheuristic and mixed integer linear programming-based exact method [52]. The stochastic parameters and uncertainties of the waste management system require new models and solution methods [53,54], as shown in the case of the collection of infectious medical waste [55]. 

Network theory can analyze waste management systems. Network theory is a part of graph theory, where a waste management network can be defined as a graph in which the nodes and/or edges represent the objects and relations. A general network model of product recycling systems includes all operations in a product recovery and waste management network for used vehicles and reuse for vehicle parts, such as collection, disassembly, refurbishing, processing, recycling, disposal, and reuse of vehicle parts [56,57]. Stream analyzes can support the design of the whole supply chain, because the analysis of data from an incoming waste stream for a waste collection center can support decision making from a logistics and technology point of view [58]. A special approach to the waste management solution includes the aspects of supplier selection. The study proposes a model that specifies the priority of products and dedicates a supplier for collecting each product [59].

**Figure 8:**
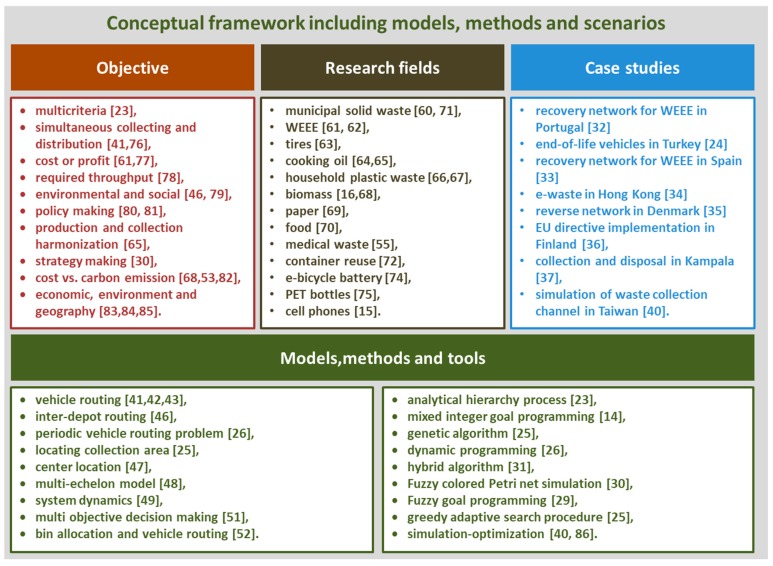
Conceptual framework of published articles [23,24,25,26,27,28,29,30,31,32,33,34,35,36,37,38,39,40,41,42,43,44,45,46,47,48,49,50,51,52,53,54,55,56,57,58,59,60,61,62,63,64,65,66,67,68,69,70,71,72,73,74,75,76,77,78,79,80,81,82,83,84,85,86].

The application field of waste management models is wide: municipal solid waste [60], wastes of electronical and electric equipment [61,62], tires [63], cooking oil [64,65], household plastic waste [66,67], biomass [16,68], paper [69], food [70], medical waste [55], municipal waste in rural region [71], container reuse [72], joint waste collection [73], e-bicycle battery [74], PET bottles [75], and cell phones [15]. The conceptual framework of published articles is shown in Figure 8.

### 2.4. Consequences of Literature Review

More than 50% of the articles were published in the last four years. This result indicates the scientific potential of the design of municipal waste management systems. The articles that addressed the optimization of waste management systems are focusing on the costs, efficiency, reliability, but none of the articles aimed to identify the optimization aspects of municipal waste collection systems from an environmental awareness and public health point of view, and none of them describe the waste management system as a cyber-physical system. Therefore, the design of waste collection systems still needs more attention and research. It was found that mathematical models and algorithms are important tools in the design and control of waste collections and treatment problems, since a wide range of models determines an optimization problem. 

The literature describes a wide range of waste collection models, but these models are usually based on conventional technologies and smart solutions that are are not taken into consideration. The transformation of conventional waste collection systems into a cyber-physical system using Industry 4.0 technologies makes the collection of more data from the system possible and makes more reliable decisions. According to that, the main focus of this research is the modelling and analysis of a cyber-physical municipal waste collection systems using the Bat Algorithm.

As a consequence, the main contributions of this article are the following: (1) model framework of cyber-physical waste collection system; (2) mathematical description of design aspects, objective functions, and time window and capacity related constraints; and, (3) computational results of the described model with various datasets and scenarios.

## 3. Model of Cyber-Physical Waste Collection System

**Figure 9:**
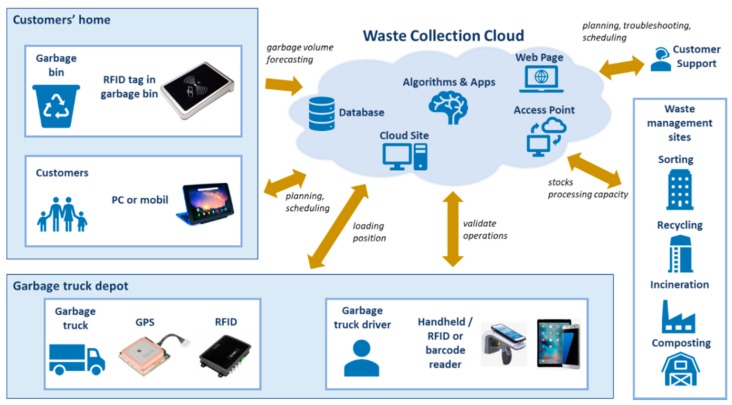
The Waste Collection Cloud and its connections with the cyber-physical waste collection system including customers, garbage trucks, waste management sites and customer support.

The research methodology of our article is based on two different types of waste collection systems. The first type of waste collection systems is represented by conventional waste collection, while the second type is a transformation of the conventional waste collection into a cyber-physical system while using Industry 4.0 technologies, smart technologies in order to increase the performance, flexibility, availability, and cost efficiency of the collection processes. The frequency of waste collection routes are established a priori, based on a fix plan [87], and they are independent of waste level in bins in conventional waste production processes. In this case, it is difficult to reach a high utilization of collection trucks, while waste collection demands are all performed. Case studies show that, in the industrial environment, containers can be equipped with level sensors and wireless communication equipment. The waste collection service provider has access to real-time information on the status of each container and the collection routes can be scheduled depending on the waste level of containers [87,88]. Another case study highlighted that, in the industrial environment, to cope with uncertainty in deposit volumes and with fluctuations due to daily and seasonal effects, an anticipatory policy is needed that balances the workload over time [89]. The cyber-physical waste collection model that is shown in Figure 9 includes customers, technological, and logistic service providers. The remote monitoring of fill level of bins makes it possible to manage the whole collection process, depending on the available volume of waste in households.

The remote monitoring of smart bins can be implemented either as real-time monitoring via Wi-Fi connection or as delayed off-line monitoring, where the collected data is transmitted through RFID readers. The data collected from smart bin is uploaded to the cloud storage. The other side of this inverse supply chain can include different waste management methods, like dumping, landfilling, composting, recycling, and incineration. However, most of the collected municipal wastes are dumped or landfilled, especially in countries with low income per capita [90], but within the frame of this model, because of their limited processing capacities, we are focusing on recycling and incineration technologies. The available processing capacities are also available in the cloud through real-time monitoring. The link between households (waste sources) and waste treatment sites is represented by garbage trucks, which are responsible for the collection of municipal waste and transportation to the treatment sites. The design and optimization of waste collection routes is performed by optimization algorithms in the waste collection cloud. The general objective function of the design of the collection process is to increase environmental awareness through the reduction of energy consumption and pollutant emission of trucks, while synchronizing the collection process with available processing capacities. Truck drivers validate all bin emptying processes with a handheld.

The municipal waste collection model includes *m* households and *n* treatment sites. There are *q* garbage trucks available at the garbage truck depot, which can perform collection service. There are defined storage capacities at the treatment sites, where the arrived waste can be stored until processing. The decision variables of this model are the following: assignment of households to routes of garbage trucks and the scheduling of garbage trucks depending on the fill level of bins and available processing capacity of treatment sites. The integration of this assignment and scheduling problem represents an NP-hard optimization problem.

With this in mind, we define the following parameters describing the layout of the cyber-physical waste collection system:

- $b_{i}$ is the position of customer *i* where i∈(1,2,…,m);
- $p_{j}$ is the position of treatment site *j* where j∈(1,2,…,n); and,
- $d_{k}$ is the position of garbage truck depot *k* where k∈(1,2,…,q).

**Figure 10:**
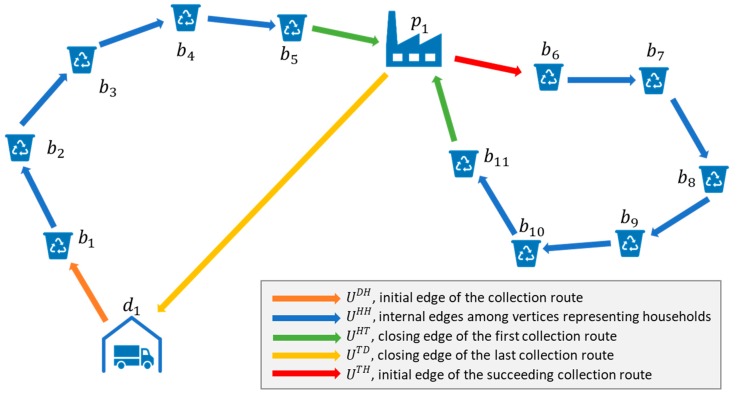
The collection route as a graph, where vertices represent the households, treatment sites, and garbage truck depots while edges correspond to transportation route.

The objective function of the problem describes the minimization of energy use of the collection process:

(1) $$\min U=U^{DH}+U^{HH}+U^{HT}+U^{TD}+U^{TH}$$

where $U^{DH}$ is the energy usage of garbage truck from garbage truck depot to the first household, where waste must be collected (initial edge of the collection route), $U^{HH}$ is the energy usage of the garbage truck among the households (internal edges among vertices representing households), $U^{HT}$ is the energy usage of the garbage truck from the last household of the collection route to the treatment site, $U^{TD}$ is the energy usage of the garbage truck from the treatment site to the garbage truck depot (closing edge of the collection route), and $U^{TH}$ is the energy usage of the garbage truck from the treatment site to the next household (initial edge of the succeeding collection route) (see Figure 10). Energy usage refers to the diesel fuel consumption of trucks, depending on the length of transportation routes and loading.

The first part of the energy usage function (1) includes the sum of energy usage of the garbage truck between the garbage truck depot and the first scheduled household. The energy usage is a function of the specific energy usage of the garbage truck without the loading and the length of route between the garbage truck depot and the first scheduled household:

(2) $$U^{DH}=\overset{q}{\underset{k=1}{\sum }}\overset{h}{\underset{\alpha =1}{\sum }}\overset{m}{\underset{i=1}{\sum }}\vartheta_{k,\alpha }\cdot l_{k,i}\left(d_{k},b_{i}\right)\cdot x_{k,\alpha ,i}^{DH}$$

where $\vartheta_{k,\alpha }$ is the specific energy usage of garbage truck α from garbage truck depot *k* to the first household of the collection route, $l_{k,i}$ is the length of the route between garbage truck depot *k* and the first household assigned to the garbage truck α, and $x_{k,\alpha ,i}^{DH}$ is the assignment matrix of the collection routes to the first household as the initial edge of the collection route.

The second part of the energy usage function (1) includes the energy usage of garbage trucks among households, excluding the route from garbage truck depot to households and routes from households to treatment sites:

(3) $$U^{HH}=\overset{q}{\underset{k=1}{\sum }}\overset{h}{\underset{\alpha =1}{\sum }}\overset{n_{\alpha }}{\underset{\beta =1}{\sum }}\overset{n_{\beta }}{\underset{\gamma =1}{\sum }}\overset{m}{\underset{i=1}{\sum }}\vartheta_{k,\alpha }^{*}\left(q_{k,\alpha ,\beta ,\gamma }\right)\cdot l_{k,\alpha ,\beta }\left(B_{k,\alpha ,\beta }\right)\cdot x_{k,\alpha ,\beta ,\gamma ,i}^{HH}$$

where $\vartheta_{k,\alpha }^{*}$ is the specific energy usage of garbage truck α from garbage truck depot *k* between households, $q_{k,\alpha ,\beta ,\gamma }$ is the loading of the garbage truck passing $\gamma^{th}$ assigned household, $l_{k,\alpha ,\beta }$ is the length of the collection route β of garbage truck α from depot *k* among assigned households, $B_{k,\alpha ,\beta }$ is the set of assigned households to the collection route β of garbage truck α from depot *k*, and $x_{k,\alpha ,\beta ,\gamma ,i}^{HH}$ is the assignment matrix of households to the collection routes.

The third part of the energy usage function (1) includes the energy use of garbage trucks from the last household of the collection route to the treatment site:

(4) $$U^{HT}=\overset{q}{\underset{k=1}{\sum }}\overset{h}{\underset{\alpha =1}{\sum }}\overset{n_{\alpha }}{\underset{\beta =1}{\sum }}\overset{n}{\underset{j=1}{\sum }}\overset{m}{\underset{i=1}{\sum }}\vartheta_{k,\alpha }^{*}\left(q_{k,\alpha ,\beta ,n_{\beta }}\right)\cdot l_{k,\alpha ,\beta ,j}\left(b_{i},p_{j}\right)\cdot x_{k,\alpha ,\beta ,n_{\beta },i}^{HT}\cdot x_{k,\alpha ,\beta ,j}^{T}$$

where $\vartheta_{k,\alpha }^{*}$ is the specific energy usage of the collection route *β* of garbage truck α from the last assigned household of the collection route to the treatment site *j*, $q_{k,\alpha ,\beta ,n_{\beta }}$ is the loading of garbage truck α passing the last household in collection route *β* from garbage truck depot *k,* $l_{k,\alpha ,\beta }$ is the length of the route between the last household assigned to the collection route *β* of garbage truck α from depot *k,* $x_{k,\alpha ,\beta ,n_{\beta },i}^{HT}$ is the assignment matrix of household to the collection routes as closing edge of the route, and $x_{k,\alpha ,\beta ,j}^{T}$ is the assignment matrix of the treatment sites to collection routes.

The fourth part of the energy usage function (1) includes the sum of energy usage of the garbage truck between the treatment sites and the garbage truck depots:

(5) $$U^{TD}=\overset{q}{\underset{k=1}{\sum }}\overset{h}{\underset{\alpha =1}{\sum }}\overset{n}{\underset{j=1}{\sum }}\overset{q}{\underset{k=1}{\sum }}\overset{h}{\underset{\alpha =1}{\sum }}\vartheta_{k,\alpha }\cdot l_{k,j}\left(d_{k},p_{j}\right)\cdot x_{k,\alpha ,n_{\beta },j}^{T}$$

where $l_{k,j}$ is the length of the route between the garbage truck depot *k* and treatment site *j* and $x_{k,\alpha ,n_{\beta },j}^{T}$ is the assignment matrix of collection routes and treatment sites.

The fifth part of the energy usage function (1) includes the sum of energy usage of the garbage truck between the treatment sites and the first assigned household of the succeeding collection route:

(6) $$U^{TH}=\overset{q}{\underset{k=1}{\sum }}\overset{h}{\underset{\alpha =1}{\sum }}\overset{n_{\alpha }}{\underset{\beta =2}{\sum }}\overset{n}{\underset{j=1}{\sum }}\overset{m}{\underset{i=1}{\sum }}\vartheta_{k,\alpha }\cdot l_{k,j,i}\left(p_{j},b_{i}\right)\cdot x_{k,\alpha ,\beta ,1}^{TH}\cdot x_{k,\alpha ,\beta -1,j}^{T}$$

The solutions of the above-described integrated scheduling and assignment problem are limited by constraints that are related to time window and capacity. Time window related constraints are defined in the case of households and treatment sites, while capacity constraints can be defined both for garbage trucks and treatment sites.

*Constraints 1*: The capacity of treatment sites is limited, so it is not allowed to exceed its treatment or processing capacity.

(7) $$\forall j:\;\overset{q}{\underset{k=1}{\sum }}\overset{h}{\underset{\alpha =1}{\sum }}\overset{n_{\beta }}{\underset{\gamma =1}{\sum }}\overset{n_{\alpha }}{\underset{\beta =1}{\sum }}\overset{m}{\underset{i=1}{\sum }}q_{i}^{HH}\cdot \left(x_{k,\alpha ,i}^{DH}+x_{k,\alpha ,\beta ,\gamma ,i}^{HH}+x_{k,\alpha ,\beta ,1}^{TH}\right)\cdot x_{k,\alpha ,\beta ,j}^{T}\leq c_{j}^{Pmax}.$$

where $c_{j}^{Pmax}$ is the upper limit of processing capacity of treatment site *j*.

*Constraints 2*: We can define a capacity limit for garbage trucks and it is not allowed to exceed the upper limit of the loading capacity:

(8) $$\forall k,\alpha ,\beta :\;\overset{n_{\beta }}{\underset{\gamma =1}{\sum }}\overset{m}{\underset{i=1}{\sum }}q_{i}^{HH}\cdot \left(x_{k,\alpha ,i}^{DH}+x_{k,\alpha ,\beta ,\gamma ,i}^{HH}+x_{k,\alpha ,\beta ,1}^{TH}\right)\cdot x_{k,\alpha ,\beta ,j}^{T}\leq c_{k,\alpha }^{Tmax}$$

where $c_{k,\alpha }^{Tmax}$ is the upper limit of processing capacity of treatment site *j* and $q_{i}^{HH}$ is the amount of waste volume in household *i* and $c_{k,\alpha }^{Tmax}$ is the loading capacity of truck α in truck depot *k*.

*Constraints 3*: We can define a timeframe for the delivery time to the treatment sites and it is not allowed to exceed the upper and lower limit of this timeframe:

(9) $$\tau_{j}^{Dmin}\leq t_{k,\alpha }^{INI}+t_{k,\alpha }^{DH}+\overset{u}{\underset{\beta =1}{\sum }}\left(t_{k,\alpha ,\beta ,j}^{HT}+t_{k,\alpha ,\beta ,j}^{TH}+\overset{n_{\beta }}{\underset{\gamma =1}{\sum }}t_{k,\alpha ,\beta ,\gamma }^{TR}+t_{k,\alpha ,\beta ,\gamma }^{LO}+t_{k,\alpha ,\beta ,\gamma }^{VA}\right)\leq \tau_{j}^{Dmax}$$

where $t_{k,\alpha }^{INI}$ is the departure time of garbage truck α from garbage truck depot *k*, $\tau_{j}^{Dmin}$ and $\tau_{j}^{Dmax}$ is the lower and upper limit of timeframe for delivery to the treatment site after the collection of waste from households, $t_{k,\alpha }^{DH}$ is the transportation time from garbage truck depot to the first household of the first collection route of garbage truck α from garbage truck depot *k*, $t_{k,\alpha ,\beta ,j}^{HT}$ is the transportation time from the last household of a collection route to the treatment site, $t_{k,\alpha ,\beta ,j}^{TH}$ is the transportation time from a treatment site to the first household of a succeeded collection route, $t_{k,\alpha ,\beta ,\gamma }^{TR}$ is the transportation time among households, $t_{k,\alpha ,\beta ,\gamma }^{LO}$ is the required loading time of the garbage truck, $t_{k,\alpha ,\beta ,\gamma }^{VA}$ is the required validation and administration time of loading at households, and *u* is the ID of collection route that is assigned to treating site *j*.

*Constraints 4*: We can define a timeframe for the scheduled emptying of bins and it is not allowed to exceed the upper and lower limit of this timeframe:

(10) $$\tau_{i}^{Emin}\leq \tau_{}^{E}\leq \tau_{i}^{Emax}$$

where $\tau_{}^{E}=\tau_{1}^{E}$ if household *i* is assigned to the first collection route as first household to be passed, $\tau_{}^{E}=\tau_{2}^{E}$ if household *i* is assigned to the first collection route as non-first household to be passed, and $\tau_{}^{E}=\tau_{3}^{E}$ if household *i* is assigned not to the first collection route, where

(11) $$\tau_{1}^{E}=t_{k,\alpha }^{INI}+t_{k,\alpha }^{DH}$$

(12) $$\tau_{2}^{E}=t_{k,\alpha }^{INI}+t_{k,\alpha }^{DH}+\overset{n_{1}}{\underset{\gamma =2}{\sum }}t_{k,\alpha ,1,\gamma }^{TR}+t_{k,\alpha ,1,\gamma }^{LO}+t_{k,\alpha ,1,\gamma }^{VA}$$

(13) $$\tau_{3}^{E}=t_{k,\alpha }^{INI}+t_{k,\alpha }^{DH}+t_{k,\alpha ,z,j}^{TH}+\overset{z-1}{\underset{\beta =1}{\sum }}\left(t_{k,\alpha ,\beta ,j}^{HT}+t_{k,\alpha ,\beta ,j}^{TH}\right)+\overset{z-1}{\underset{\beta =1}{\sum }}\overset{n_{\beta }}{\underset{\gamma =1}{\sum }}(t_{k,\alpha ,\beta ,\gamma }^{TR}+t_{k,\alpha ,\beta ,\gamma }^{LO}+t_{k,\alpha ,\beta ,\gamma }^{VA})$$

where $\tau_{j}^{Dmin}$ and $\tau_{j}^{Dmax}$ is the lower and upper limit of timeframe for delivery to the treatment site after the collection of waste from households, $t_{k,\alpha }^{DH}$ is the transportation time from garbage truck depot to the first household of the first collection route of garbage truck α from garbage truck depot *k*, $t_{k,\alpha ,\beta ,j}^{HT}$ is the transportation time from the last household of a collection route to the treatment site, $t_{k,\alpha ,\beta ,j}^{TH}$ is the transportation time from a treatment site to the first household of a succeeded collection route, $t_{k,\alpha ,\beta ,\gamma }^{TR}$ is the transportation time among households, $t_{k,\alpha ,\beta ,\gamma }^{LO}$ is the required loading time of the garbage truck, and $t_{k,\alpha ,\beta ,\gamma }^{VA}$ is the required validation and administration time of loading at households.

There are two types of decision variables: the decision variables of the assignment problem are binary matrices, while the decision variable of the scheduling problem is a matrix with real values. The assignment matrices (14) defines the assignment of emptying smart household bins to collection routes

(14) $$x_{k,\alpha ,i}^{DH}\in \left(0,1\right)\wedge x_{k,\alpha ,\beta ,\gamma ,i}^{HH}\in \left(0,1\right)\wedge x_{k,\alpha ,\beta ,n_{\beta },i}^{HT}\in \left(0,1\right)\wedge x_{k,\alpha ,\beta ,1}^{TH}\in \left(0,1\right)\wedge x_{k,\alpha ,\beta ,j}^{T}\in \left(0,1\right)$$

The scheduling matrix of the collection routes is represented by the $t_{k,\alpha }^{INI}$ value, which defines the departure time of the first route of garbage truck α from garbage truck depot *k*:

(15) $$t_{k,\alpha }^{INI}\in \mathbb{R}$$

## 4. Binary Bat Optimization Algorithm

Bat algorithm (BA) is a representation of particle swarm optimization (PSO). Particle swarm optimization algorithms are based on the swarm behavior of animals. There are a wide range of swarming behavior-based algorithms in the literature, like artificial bee colony algorithm [91], fish school search [92], bat algorithm [93], krill herd [94], black hole optimization [95], big bang big crunch algorithm [96], gravitational search [97], firefly algorithm [98], flower pollination algorithm [99], ant-based routing algorithm [100], and fruit fly optimization [101]. Bats are mammals of the order Chiroptera. They are the only mammals that are naturally capable of true and sustained flight. Bats are more maneuverable than birds. Microbats are using echolocation to find food, avoid obstacles, and to locate their roosting crevices in the dark [102]. The bat algorithm is based on the swarming of microbats. The swarming process of microbats is based on the updating process of their position and velocity in the multidimensional search space that represents the multi-dimensional optimization problem. We assign a frequency, a wavelength, and a pulse emission rate representing its echolocation to each microbat. We can calculate the frequency each microbat, as follows:

(16) $$f_{i}=f_{min}+\mu \left(f_{max}-f_{min}\right)$$

where $f_{i}$ is generated between a uniform frequency interval between $f_{min}$ and $f_{max}$ and μ∈[0,1] is a uniformly distributed value.

We can calculate the velocity of each microbat in iteration step t, as follows:

(17) $$v_{i}\left(t\right)=v_{i}\left(t-1\right)+f_{i}\left(x_{i}\left(t\right)-x^{OPT}\left(t\right)\right)$$

where $v_{i}\left(t\right)$ is the velocity of microbat *i* in iteration step *t* and $x^{OPT}$ is the global best solution in iteration step *t*.

We can calculate the position of each microbat in iteration step *t,* as follows:

(18) $$x_{i}\left(t\right)=x_{i}\left(t-1\right)+v_{i}\left(t\right)$$

where $x_{i}\left(t\right)$ is the position of microbat *i* in iteration step *t*.

However, the bat algorithms are suitable for the solution of continuous optimization problems, but there are mathematical methods to solve discrete problems with bat optimization, for example, by the use of the sigmoid function [103]. We can calculate a sigmoid function describing a characteristic “S”-shaped curve or sigmoid curve from the microbats’ velocity; thereafter it is possible to update the binary position, as follows:

(19) $$x_{i}\left(t\right)=\{\begin{array}{c} 0\;if\;\theta <S\left(v_{i}\left(t\right)\right)\\ 1\;if\;\theta \geq S\left(v_{i}\left(t\right)\right) \end{array}$$

where $S\left(v_{i}\left(t\right)\right)$ is the sigmoid function calculated from the velocity of microbats:

(20) $$S\left(v_{i}\left(t\right)\right)=\frac{e^{-v_{i}\left(t\right)}}{1+e^{-v_{i}\left(t\right)}}$$

After the global search, we can perform a local search. The local search possibility is influenced by the pulse emission rate. If $\xi >r_{i}\left(t\right)$, then a local search is performed. The local search means the update of a selected best solution after global search:

(21) $$x^{OPT}\left(t\right)=x^{OPT}\left(t-1\right)+\varpi L\left(t\right)$$

where ϖ∈[0,1] is a uniformly distributed random value and L(t) is the loudness function, which can be calculated as follows:

(22) $$L_{i}\left(t\right)=L_{i}\left(t-1\right)\alpha$$

The pulse emission rate must be updated after each local search based on the following equation:

(23) $$r_{i}\left(t\right)=r_{i}\left(0\right)\left(1-e^{-\gamma t}\right)$$

where α and γ are predefined constants [103].

Table 1 shows the results of performance analysis of bat algorithm, where BA is compared with other heuristic algorithms [104]. We used benchmarking functions [105] to test the error values of the heuristic algorithms in 50 iteration steps.

As the performance of heuristic algorithms that were measured with benchmarking functions shows, the bat algorithm is also suitable for the solution of NP-hard optimization problems. Within the frame of the next chapter, the application of bat algorithm is shown in the case of cyber-physical waste management systems.

## 5. Results and Discussions: Scenario Analysis of Cyber-Physical Waste Collection Systems Focusing on Environmental Awareness

Within the frame of this chapter, two scenarios will demonstrate the application possibilities of the above-described mathematical model and validate the applied heuristic optimization algorithm with different data sets. The optimization algorithm (1–15) takes care of a fleet with different trucks and we can also choose different bin sizes, but the scenarios are simplified to make the examples as perspicuous as possible. Table 2 demonstrates the generated waste volume in each household for a time period of 10 days. We use these volumes to analyze the various strategies of waste management system in the case of traditional operation and in the case of a cyber-physical system. The processing capacity of the treatment site is 450 volume units (VU) per day, the loading capacity of the garbage truck is 930 VU, and the loading volume of a bin is 120 VU. In this case, we only use the above-described heuristic optimization for the routing of garbage trucks because the scheduling is a static sequenced scheduling and there is no available information on bins’ loading levels.

### 5.1. Scenario 1: Periodical Collection Routes in Conventional Waste Management System

In the case of the first scenario, the waste management system is performed as a traditional collection system without the use of IoT technologies. The coordinator of the waste management system has no real-time information regarding the waste level in the households’ bins; therefore, the emptying process of bins is scheduled as a sequence of periodic collection routes. Table 3 shows the calculated generated waste in household in the case of periodical collection routes every two days.

**Figure 11:**
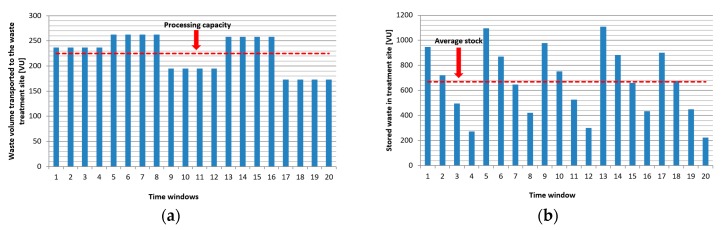
(**a**) Average waste volume between two supply transported to the waste treatment site in Scenario 1. The waste supply is not synchronized with the processing capacity, because the supplied volume of waste exceeded the required amount in the time windows 1 to 8 and 13 to 16, while not reached the required amount in time windows 9 to 12 and 17 to 20; and, (**b**) Waste inventory at the treatment site (average = 668 VU, max = 1109 VU).

The numbers in the red boxes show the households and time windows where the generated waste exceeded the loading capacity of bins. It means that the garbage trucks collected the waste later and the waste bins were full in the following cases: in the fourth time window three customers, in the eighth time window one customer, and in the sixteenth time window four customers were not sought out in time, so the availability of the waste collection service level was 84% and the customers stored 94 VU waste outside the bin within the frame of the time span of the analysis. Figure 11a shows the waste volume that was transported to the waste treatment site. Because of the lack of information of bins’ loading level and the static, periodic scheduling, or collection routes, the transported volume and the available processing capacity are not synchronized. As Figure 11b demonstrates, storage capacity must be available for the storage of waste to not be processed.

**Figure 12:**
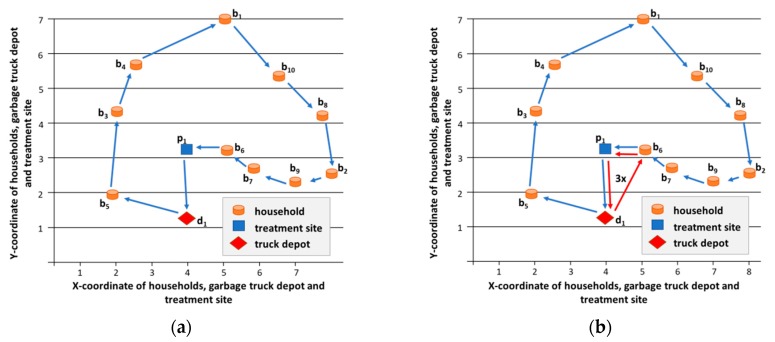
Collection routes: (**a**) Original route. (**b**) If overload of garbage truck is not allowed, then three additional collection routes must be inserted.

The routing algorithm calculated the *depot-b_5_-b_3_-b_4_-b_1_-b_10_-b_8_-b_2_-b_9_-b_7_-b_6_-p_1_-depot* sequence as the optimal collection route, where the total length of the five collection routes was 103.056 km with a diesel consumption of 32.98 liters. This diesel consumption means 86698 g CO_2_ emission, 2.63 g SO_2_ emission, 72.56 g CO emission, 39.58 g HC emission, 392.44 g NO_X_ emission, and 3.298 g PM emission. From Table 2, we can calculate that the five sequenced collection routes will collect 947, 1049, 781, 1032, and 691 VU. It means that, in this scenario, the garbage trucks are overloaded in three cases: fourth, eighth, and sixteenth time window and the total overload is 238 VU. If overload is not allowed for garbage trucks, then the collection must be rescheduled (see Figure 12).

After rescheduling of the collection routes within the frame of the time span of analysis, the length of the total collection route was increased by 15.36 km with an additional diesel consumption of 4.92 liters. This additional diesel consumption, which was caused by the additional *depot- b_6_-p_1_-depot* routes, increases the CO_2_ emission with 12935 g, the SO_2_ emission with 0.39 g, the CO emission with 10.82 g, the HC emission with 5.90 g, the NO_X_ emission with 58.55 g, and the PM emission with 0.49 g. As a summary of Scenario 1, Table 4 shows the calculated emission of collection routes to demonstrate the environmental impact.

### 5.2. Scenario 2: Dynamic Collection Route Scheduling in a Cyber-Physical Waste Management System

In the case of the second scenario, the waste management system is performed as a cyber-physical system, where Industry 4.0 technologies and paradigms support the optimal operation of the processes. Real-time information on the loading level of bins is available in the waste management cloud and it is possible to schedule the routes and assign the households to collection routes so that the service level of the whole waste management system can be increased. Within the frame of this scenario, we are calculating the generated waste amount that is shown in Table 2.

**Figure 13:**
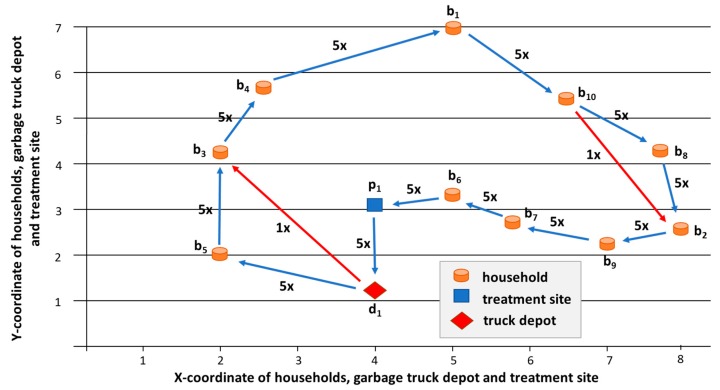
Collection routes of Scenario 2: the five similar periodic collection routes were changed. Both the scheduling and the sequence of households were rescheduled to reduce the emission to increase the environmental awareness of the collection process. The optimization algorithm resulted in a emission reduction of 14.78%.

The enhanced routing and scheduling algorithm calculated three various routes to collect all of the generated waste from households (see Figure 13).

The second, fourth, and fifth routes are *depot-b_5_-b_3_-b_4_-b_1_-b_10_-b_8_-b_2_-b_9_-b_7_-b_6_-p_1_-depot* sequences, where the total length of these three collection routes was 61.83 km with a diesel consumption of 19.79 liter. The first collection route is a *depot-b_5_-b_3_-b_4_-b_1_-b_10_- b_2_-b_9_-b_7_-b_6_-p_1_-depot* sequence, where the total length of this collection route is 20.37 km with diesel consumption of 6.52 liter. The third route is a *depot-b_3_-b_4_-b_1_-b_10_-b_8_-b_2_-b_9_-b_7_-b_6_-p_1_-depot* sequence, where the total length of this collection route was 19.91 km with a diesel consumption of 6.37 liters. The diesel consumption of the total collection routes within the time span of the analysis means 85911 g CO_2_ emission, 2.61 g SO_2_ emission, 71.89 g CO emission, 39.21 g HC emission, 388.87 g NO_X_ emission, and 3.26 g particulate matter (PM) emission (see Table 5).

**Figure 14:**
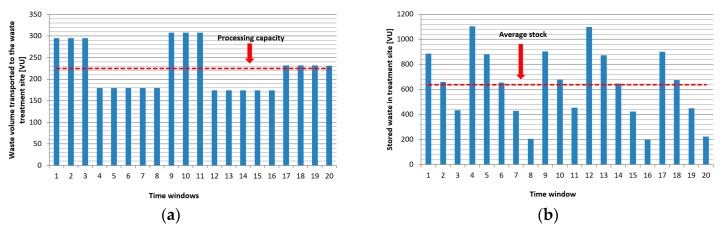
(**a**) Average waste volume between two supply transported to the waste treatment site in Scenario 2. The waste supply is not synchronized with the processing capacity, because the supplied volume of waste increased the required amount in the time windows 1 to 3, 9, to 11 and 17 to 20, while not reaching the required amount in time windows 4 to 8 and 12 to 16; (**b**) Waste inventory at the treatment site (average = 639 VU, max = 1105 VU).

Figure 14a shows the waste volume that was transported to the waste treatment site. Within the frame of this scenario, the collection process is optimized from an environmental awareness point of view, including the minimization of emission of garbage trucks and the transported volume and the available processing capacity is not synchronized. As Figure 14b demonstrates, the storage capacity must be available for the storage of waste that is caused by asynchronous supply.

The above-described scenarios validated the presented cyber-physical model of a waste management system in the collection of municipal waste and justify the fact that the application of Industry 4.0 technologies to transform a traditional waste management system to a cyber-physical system strongly influences the performance of the whole supply process, while sustainability and environmental awareness are also taken into consideration in the form of minimization of energy consumption and emission.

To summarize, the proposed optimization model that was based on the binary bat algorithm makes the analysis of the impact of scheduling and assignment on economical and ecological (public health) aspects, where public health is influenced by the emissions that are caused by the collection process, possible.

The analyses of both scenarios show that the uncertainty in waste volume can be handled with the application of level sensors in bins and wireless communication equipment. Waste collection service providers can access the collected data in the waste management cloud and additional optimization opportunities will be available. As the scenarios show, the optimization of waste collection routes, including time- and capacity-related constraints lead to the decreased length of transportation routes, decreased energy consumption of waste collection trucks, and decreased greenhouse gas emission.

As the findings of the literature review show, the articles that addressed the analysis of waste management systems are focusing on the cost optimization and efficiency problems of conventional waste management systems, but none of the articles aimed to identify the potential of IoT solutions to increase the environmental awareness of the waste collection process. The comparison of our results with those from other studies shows that the design and operation of waste management systems in an Industry 4.0 environment, and the transformation of a traditional waste management system to a cyber-physical system, still need more attention and research. The reason for this is that, in the case of large-sized stochastic systems, like waste collection systems, the availability of real-time information on generated waste volume, available processing, transportation, and material handling capacity must be taken into consideration.

In spite of the small size of the demonstrated problems, these results show that the proposed method using heuristic optimization performs better than the traditional formal models to evaluate waste management related problems. Traditional formal models are generally focusing on cost-based optimization of traditional municipal solid waste collection: the effective service network design in the Nanjing Jiangbei new area is performed with an emphasis on minimizing the annual operation costs, while the number, size, and location of refuse transfer stations is optimized [107]. In our opinion, the policy-makers can influence the development direction of waste management systems. There must be some policy interventions to support the technological investment, especially from an Industry 4.0 and IoT point of view [108]. However, the determining factors of a successful implementation of a green cyber-physical waste management system are based on used Industry 4.0 technologies and IoT solutions, but there are other influencing factors that are to be taken into considerations, like the environmental consciousness of the population, the level of environmental culture in the region, generations’ characteristics [109], or the legal regulations determining recycling and waste management operations.

As the mathematical model (see Equations (1)–(15)) shows, the technological aspects can be taken into consideration in the model and they can be described by operation and decision-making strategies. The model was intended to assist operation managers in deciding optimum strategic and tactical plans for operation according to the available real-time information on the generated waste amount and the available technological and logistic capacities.

## 6. Discussion 

Within the frame of this research work, the authors used a binary bat algorithm-based optimization model, which makes the analysis of the performance of traditional and cyber-physical waste management system form collection processes point of view possible. More generally, this paper focused on the mathematical description of the framework of waste management systems, including households as waste sources, treatment sites, and collection processes. Why is so much effort being put into this research? The role of waste management systems become more and more important, because it is a priority to move from reliance on waste dumps that offer no environmental protection, to waste management systems that retain useful resources within the economy and its operation is green and does not endanger the public health through environmental pollution [110].

However, there are also limitations of the study and the described model that shows directions for further research. Our model takes care of a fleet with different trucks, because the software makes it possible to set the loading capacity of each truck. We can also choose the bin size for each household, but it is indifferent from waste volume point of view, because smart bins are able to detect the level of waste in the bin and then calculate the volume depending on the bin’s type (edge computing). Within the frame of this model, the stochastic parameters of the environment and other uncertainties are not taken into consideration. In further studies, the model can be extended to a more complex model, including a description of uncertain parameters with fuzzy models and another direction is to apply fuzzy colored Petri net simulation [111,112]. Second, this study only considered a single echelon recycling/reuse process, but it is also possible to model multi-echelon reuse and recycling technologies [113,114], including disassembly or shredding technologies. Future research models can also include theses aspects. The European Union (EU) policies on waste are based on the waste hierarchy and recycling plays an important role. In the EU waste framework directive, there are obligations that some waste fractions have to be collected separately at the source, and the collection systems in many EU countries are based on the collection of several waste fractions. Within the frame of our future research, we are going to focus on the waste collection as one entity in a holistic perspective, and have a focus on all fractions that have to be collected, not only one fraction, as in our model discussed. The discussed model can be extended to a multi-fraction collection model, because the mathematical description can be transformed into a model, which sees the collection of different types of waste as one entity. The customers’ network frequently resist changes as group of staff communities and the introduction of customer relationship management services can lead to significant changes [115]. This should be also considered in future research.

Regarding WEEE or e-waste management, there are some new and innovating publications on models and methods in China or Hongkong, which should also be taken into consideration in our future research. Researchers found that collection companies in China perform two different intelligent collections: human-human interaction collection and human machine interaction collection. Comparative advantages were found in organization, trade, data accumulation, and profit-making sources. However, intelligent collection in China is still at an early stage, but its potential for a sustainable business model needs to be further explored [116,117]. However, the application of smart collection systems is widening, but the public willingness to pay and participate in domestic waste management influences the performance of collection, especially in rural areas [118]. As the “Internet+Recycling” model shows, the intelligence of Chinese solutions is based on Internet of Things solutions. This waste collection model enables individuals to arrange collection appointment through various online platforms and then the collectors pick up the waste on-site [119]. The quantity of collected waste influences the logistics processes of waste management, while the quality has a great impact on the waste processing technologies. Studies show that the incinerability index is different in different economic groups, which means that a different proportion of waste processing technologies must be used [120]. The processing technologies of wastes, especially in the case of wastes of electric and electronic equipment, where the disassembly and remanufacturing operations are performed, and further studies are required to describe the influencing factors of productivity, like in the case of hard machining [121]. The attitude behavior-condition theory is a suitable method to support decision making processes in waste management and to analyze the decision-making mechanism of residents’ HSW disposal behaviors [122]. Leadership style is an important factor that affects the enhancement of organizational performance and employee’s job performance [123], which has significant impact on the global processes of the waste management system, therefore it should be also considered in our future research.

## 7. Conclusions

The added value of the paper is the description of the mathematical model of the traditional and cyber-physical waste management system, which makes the description of the impact of Industry 4.0 technologies, like RFID, cloud and fog computing, big data analysis when developing and operating them possible. The scientific contribution of this paper for researchers in this field is the mathematical modelling and optimization of waste collection processes based on binary bat algorithm-based optimization. The results can be generalized, because the model can be applied for different fields of waste management systems from the wastes of electrical and electronic equipment (WEEE) to biomass or medical waste.

## Figures and Tables

**Table 1:** Error values of bat algorithm (BA) and other three heuristic algorithms ^1^.

Benchmarking Function [102]	BHO ^2^	GA ^3^	HS ^4^	BA ^5^
Ackley	3.66 × 10^−7^	4.67 × 10^−6^	1.28 × 10^−7^	1.84 × 10^−8^
Bukin	2.45 × 10^−6^	5.45 × 10^−7^	9.08 × 10^−7^	4.57 × 10^−7^
Cross-in-tray	8.55 × 10^−9^	7.32 × 10^−9^	6.98 × 10^−8^	1.04 × 10^−6^
Easom	1.18 × 10^−5^	2.09 × 10^−4^	8.18 × 10^−9^	6.73 × 10^−9^
Eggholder	5.50 × 10^−7^	3.12 × 10^−7^	1.98 × 10^−8^	8.11 × 10^−8^
Himmelblau	5.79 × 10^−8^	2.25 × 10^−6^	1.05 × 10^−8^	9.42 × 10^−7^
Lévi	1.20 × 10^−6^	7.34 × 10^−8^	3.12 × 10^−8^	6.54 × 10^−5^
Matyas	9.12 × 10^−8^	1.78 × 10^−7^	6.70 × 10^−9^	1.14 × 10^−7^
Modified sphere	2.21 × 10^−8^	1.93 × 10^−6^	2.40 × 10^−8^	4.25 × 10^−7^
Three hump camel back	1.51 × 10^−6^	4.17 × 10^−8^	7.79 × 10^−10^	5.79 × 10^−9^

^1^ After 50 iteration steps. The results of BA are compared with results in [101]. ^2^ BHO = Black Hole Optimization. ^3^ GA = Genetic Algorithm. ^4^ HS = Harmony Search. ^5^ BA = Bat Algorithm.

**Table 2:** Input parameters of scenarios: Generated volume of waste in households ^1^.

HH ^2^	Sequence of Time Windows																			
	1	2	3	4	5	6	7	8	9	10	11	12	13	14	15	16	17	18	19	20
**1**	15	19	14	23	39	40	36	28	11	36	18	11	19	33	37	27	40	27	25	11
**2**	10	35	24	19	40	30	26	0	26	19	32	20	7	27	24	40	10	4	2	21
**3**	40	25	4	38	39	6	33	30	9	14	14	15	17	13	13	6	1	25	18	6
**4**	36	12	37	37	39	38	11	12	37	4	7	18	33	10	33	2	14	30	20	38
**5**	27	38	21	35	35	31	22	17	18	23	1	14	3	23	29	27	23	8	24	14
**6**	1	3	36	35	35	30	15	39	8	37	12	21	36	26	39	32	23	7	3	38
**7**	33	1	29	35	29	16	29	35	31	30	23	0	37	21	41	28	8	32	9	9
**8**	21	1	31	9	16	13	32	36	35	20	23	34	42	34	32	35	31	8	11	22
**10**	33	10	4	22	38	28	30	14	33	15	1	22	32	40	27	32	20	6	5	30

^1^ The generated waste volume is given in volume units (VU). ^2^ HH = Households.

**Table 3:** The cumulative volume of generated waste in households ^1^.

HH ^2^	Sequence of Time Windows																			
	1	2	3	4	5	6	7	8	9	10	11	12	13	14	15	16	17	18	19	20
**1**	15	34	48	71	39	79	115	143	11	47	65	76	19	52	89	116	40	67	92	103
**2**	10	45	69	88	40	70	96	96	26	45	77	97	7	34	58	98	10	14	16	37
**3**	40	65	69	107	39	45	78	108	9	23	37	52	17	30	43	49	1	26	44	50
**4**	36	48	85	122	39	77	88	100	37	41	48	66	33	43	76	78	14	44	64	102
**5**	27	65	86	121	35	66	88	105	18	41	42	56	3	26	55	82	23	31	55	69
**6**	1	4	40	75	35	65	80	119	8	45	57	78	36	62	101	133	23	30	33	71
**7**	33	34	63	98	29	45	74	109	31	61	84	84	37	58	99	127	8	40	49	58
**8**	21	22	53	62	16	29	61	97	35	55	78	112	42	76	108	143	31	39	50	72
**9**	33	43	47	69	38	66	96	110	33	48	49	71	32	72	99	131	20	26	31	61
**10**	37	69	97	134	28	31	57	62	7	40	56	89	10	35	68	75	39	52	54	68

^1^ The generated waste volume is given in volume units (VU). ^2^ HH = Households. The sequences in the time window means half days (Monday am = 1, Monday pm = 2, Tuesday am = 3, Tuesday pm = 4, etc.).

**Table 4:** Calculated emission of collection routes.

Routes	Route Length	Emission					
		CO_2_	SO_2_	CO	HC	NO_X_	PM
**Specific emissions in g/liter fuel consumption [106]**	-	2629	0.08	2.2	1.2	11.9	0.1
**Collection route with overloaded truck**	103.056	86698	2.63	72.56	39.58	392.44	3.29
**Collection route without overloaded truck**	118.420	99633	3.02	83.38	45.48	450.99	3.78
**Additional routes to eliminate overloading**	15.360	12935	0.39	10.82	5.90	58.55	0.49

^1^ The generated waste volume is given in volume units (VU). ^2^ HH = Households.

**Table 5:** Calculated emission of collection routes in Scenario 2.

Routes	Route Length	Emission					
		CO_2_	SO_2_	CO	HC	NO_X_	PM
**2nd, 4th and 5th collection routes without overloaded truck**	61.83	52019	1.58	43.53	23.74	235.46	1.97
**1st collection route without overloaded truck**	20.37	17144	0.52	14.35	7.82	77.60	0.65
**3rd collection route without overloaded truck**	19.91	16747	0.51	14.01	7.64	75.80	0.63
**Total collection route without overloaded truck**	102.11	85911	2.61	71.89	39.21	388.87	3.26
